# MXene‐Based Nanocomposites for Supercapacitors: Fundamentals and Applications

**DOI:** 10.1002/smtd.202401751

**Published:** 2025-04-29

**Authors:** Nanasaheb M. Shinde, Martin Pumera

**Affiliations:** ^1^ Advanced Nanorobots & Multiscale Robotics Faculty of Electrical Engineering and Computer Science VSB – Technical University of Ostrava 17. listopadu, 2172/15 Ostrava 70800 Czech Republic

**Keywords:** 2D materials, energy storage, MAX

## Abstract

MXene‐based nanocomposite materials with other 2D materials have made a large impact in the field of energy storage, particularly in the area of supercapacitors. Combining conductive 2D MXene with other 2D materials, such as transition metal oxide, transition metal dichalcogenides, and layered double hydroxide, improves the electrochemical energy storage properties of resulting MXene‐based nanocomposites. The interface of MXene and 2D nanocomposite materials allows an improved electrochemical performance for energy storage applications. In this review, state‐of‐the‐art research progress in 2D/2D MXene‐based nanocomposite synthesis, structural and morphological properties, and electrochemical performance for supercapacitors is explored. 2D MXene nanocomposites electrochemical properties in terms of specific capacitance, energy, power densities, and stability are discussed. This study shows that this rapidly developing field has an important impact on the next‐generation supercapacitor.

## Graphical abstract

MXene‐based nanocomposites with 2D materials like transition metal oxides, transition metal chalcogenides, and layered double hydroxides improve supercapacitor performance. Here it reviews how these composites facilitate high capacitance, power density, and cycling stability, advancing next‐generation energy storage.

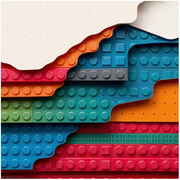

## 1 Introduction

The burgeoning interest in 2D materials within the realm of electrochemical devices stems from their remarkable properties and is spurred by current research demands. This surge has propelled an exploration into their potential, particularly in the context of supercapacitor devices, leveraging their distinctive stacked 2D structure.^[^ ^1^, ^2^, ^3^ ^]^ 2D structured materials like transition metal oxide (TMO), transition metal dichalcogenides (TMDs), and layered double hydroxide (LDH) and MXene have been studied in recent years.^[^ ^4^, ^5^, ^6^, ^7^ ^]^ The above‐mentioned 2D materials have been prepared using different top‐down or bottom‐up methods.^[^ ^8^, ^9^, ^10^, ^11^, ^12^, ^13^, ^14^, ^15^, ^16^, ^17^, ^18^, ^19^, ^20^, ^21^ ^]^ 2D materials show important properties for electrochemical substances, *e.g*., thin nanosheets of 2D materials with a large surface area and good electronic conductivity, flexibility, strength, and stability.^[^ ^22^, ^23^, ^24^, ^25^, ^26^, ^27^, ^28^ ^]^ MXene, first identified in 2011,^[^ ^29^, ^30^ ^]^ has sparked research interest due to its characteristics and good performance in a broad range of applications. MXene comprises a promising collection of 2D materials, including transition metal carbides, nitrides, and carbonitrides.^[^ ^31^, ^32^ ^]^ MXene exhibits very good structural, electrical, and electrochemical characteristics.^[^ ^33^, ^34^, ^35^ ^]^ The basic chemical structure is M_y_ *_+_*_1_X*_y_* T*_x_*, where M = transition metal, X = C/N/C_x_N_y_, and T = −OH, −O, −F, etc.^[^ ^36^ ^]^ MXene has excellent conductivity and surface area, and variable oxidation states, which is advantageous for supercapacitors.^[^ ^37^, ^38^ ^]^ Although pristine MXene offers outstanding electrochemical properties when it is employed for real commercial applications for longer periods it faces some issues, e.g., re‐stacking of its nanosheet structures.^[^ ^39^ ^]^ Therefore, direct use of 2D MXene has limitations, including lower capacitance, reduced stability, a non‐reactive nanostructure, and hindered electrolyte diffusion due to re‐stacking.^[^ ^40^ ^]^ To seek a solution to these issues, 2D MXene‐based nanocomposite materials have been highly explored in recent years for their large capacity, high energy density, rate performance, stability contribution, and higher supercapacitance.^[^ ^41^ ^]^ MXene has high electronic conductivity and when combined with other materials this increases its performance.^[^ ^38^, ^39^ ^]^

**Figure 1:**
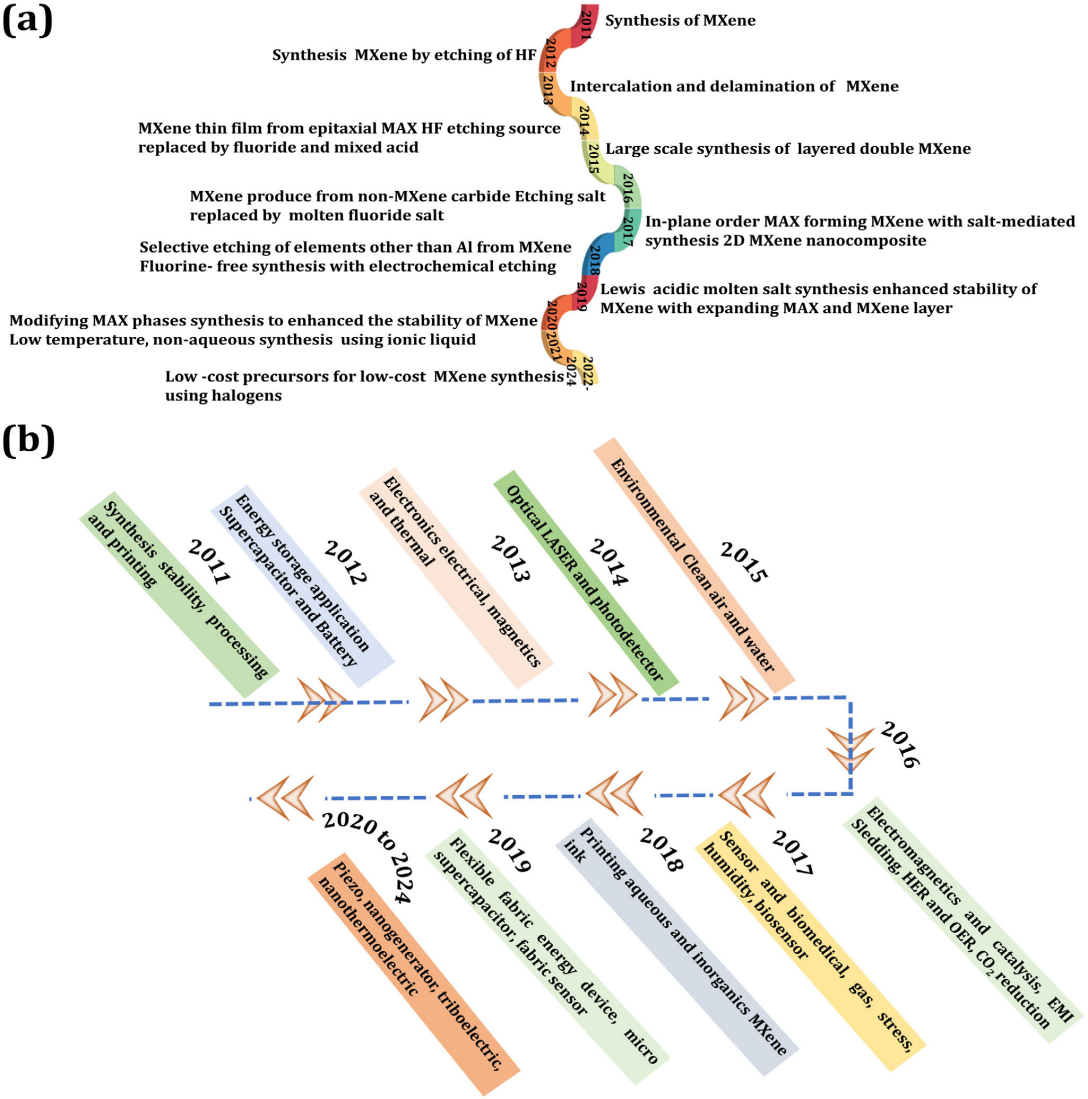
a) History of MXene synthesis during the last 12 years. b) Various major applications of MXene‐based nanocomposites.

MXene nanocomposites demonstrate increased overall electrochemical properties.^[^ ^3^, ^4^, ^10^ ^]^ Thousands of articles have been published related to MXene and its composites across a broad range of research fields. **Figure** **1**a describes the synthesis progress of MXene over the last 12 years. Figure 1b highlights the most important points of MXene nanocomposite application in various fields. In this review, focused on supercapacitor systems, we first discuss the preparation methods and several important properties of MXene. Subsequently, electrochemical properties of MXene‐based nanocomposites with other 2D materials, such as transition metal oxide (TMO), transition metal dichalcogenides (TMDs), and layered double hydroxide (LDH) are discussed. A comprehensive analysis of research on MXene‐based nanocomposites for use in supercapacitors. In the end, an outline of the future perspectives and challenges are summarized for next‐generation energy storage and other applications of MXene nanocomposites.

## 2 MXene Synthesis

**Figure 2:**
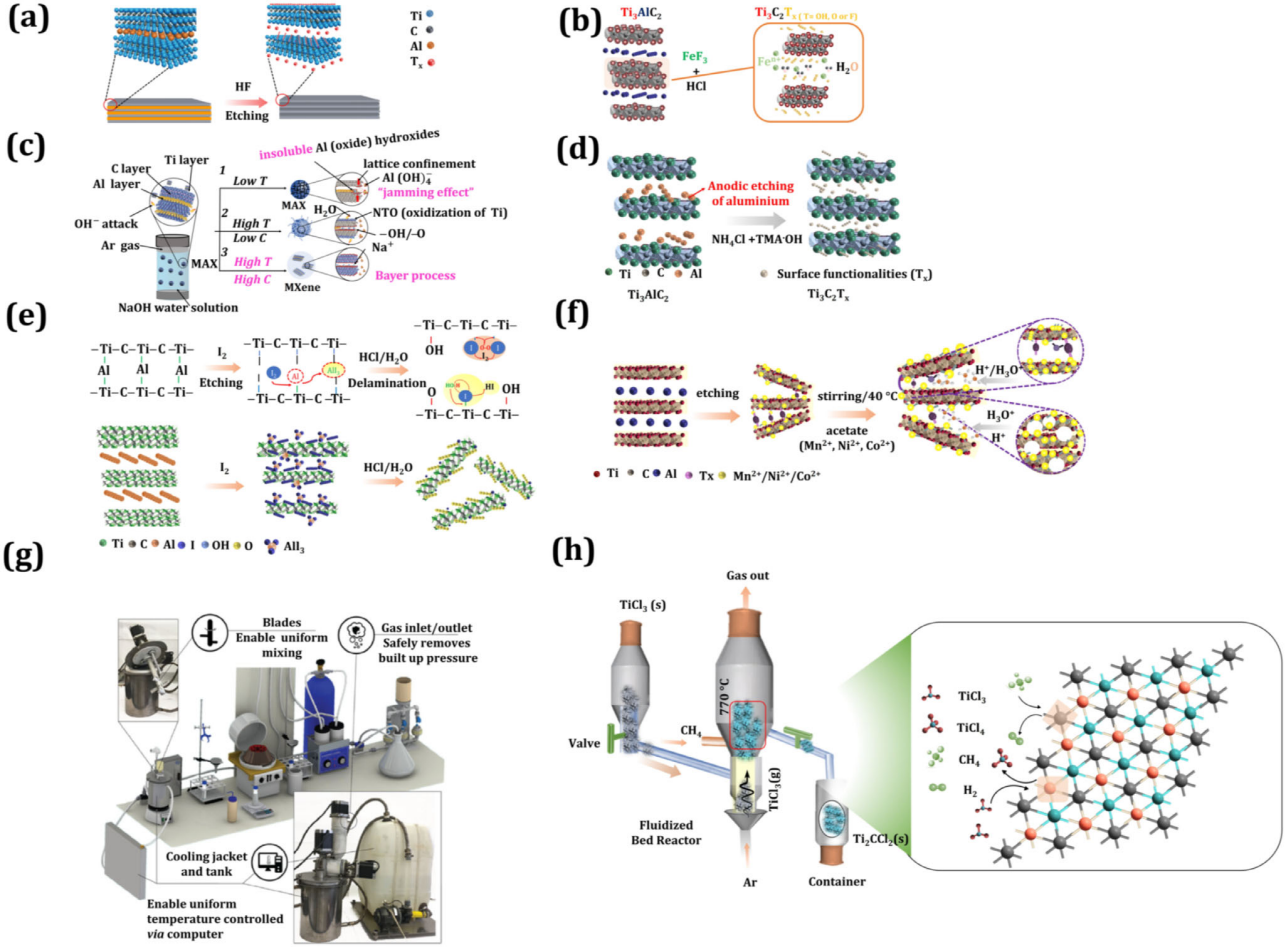
MXene synthesis approaches. Top‐down approaches: a) conventional HF etching process for MXene synthesis wherein Ti_3_AlC_2_ was added into hydrofluoric (HF) acid (Re‐draw from reference paper).^[^ ^40^ ^]^ HF‐free etching MXene synthesis process included b) acid/salt etching process in which a combined FeF_3_ and HCl solution was used for MXene synthesis (Re‐draw from reference paper),^[^ ^41^ ^]^ c) fluorine‐free strategies in the presence of NaOH–water solution with different experimental conditions: (1) at lower temperature: formation of insoluble Al (oxide) hydroxide; (2) at higher temperature and low NaOH concentrations: dissolve Al (oxide) hydroxide in NaOH; (3) higher temperatures and higher NaOH concentrations: completely dissolve the Al(oxide) hydroxides in NaOH solution (the Bayer process) (Re‐draw from reference paper),^[^ ^42^ ^]^ d) fluorine‐free anodic etching in binary solution of NH_4_Cl and tetramethylammonium hydroxide with applied potential of +5 V,) (Re‐draw from reference paper)^[^ ^43^ ^]^ e) fluorine‐free MXene etching using iodine in anhydrous acetonitrile solution followed by delamination in HCl solution (Re‐draw from reference paper),^[^ ^45^ ^]^ f) ion‐induced etching, e.g., HF etching of MXene followed by stirring in different metal ion solutions (*e.g*., Mn, Ni, Co) (Re‐draw from reference paper),^[^ ^46^ ^]^ and g) large‐scale MXene synthesis process, including several steps and an internal system connected with a cooling jacket and tank to allow uniform temperature controlled via a programmable computer. There is automatics attached to gas, drying process, and adjustable pressure, as a result, permit homogeneous feeding of MAX powder. Reproduced with permission.^[^ ^47^ ^]^ Copyright 2020, John Wiley and Sons. Bottom‐down approaches h) gas‐phase chemical vapor deposition (CVD) synthesis in which TiCl_3_ powders were added to the reactor and then converted into a gaseous precursor, TiCl_3_(g). Additionally, treatment in an argon (Ar) atmosphere, followed by a reaction with methane at 770 °C results in the formation of Ti₂CCl₂ powders (Re‐draw from reference paper).^[^ ^48^ ^]^

MXene (Ti_3_C_2_T*_x_*) is typically derived from the MAX phase, employing aqueous HF as an etchant for the removal of the Al layer at ambient conditions.^[^ ^33^, ^34^, ^35^, ^36^ ^]^ It should be underlined that the stacked layers are selectively etched using HF to generate a larger area for Li adsorption and desorption processes over the surface of the MAX phase. However, the removal of Al depends on many factors, including acid concentration, temperature, time, and so on. Enhancing control over etching conditions serves as a crucial rationale for enabling large‐scale production and minimizing the degradation of 2D nanosheets during the etching process.^[^ ^37^, ^38^ ^]^ This etching process arises from top‐down methods in which the Al element is removed from the MAX phase using the wet chemical method. This is the most normally used and established method. In the subsequent section, we briefly describe the different top‐down methods. The preparation entails utilizing an aqueous solution containing fluoride ions (**Figure** **2**a) to modify the surface groups of MXene, namely, O, OH, and F, referred to as T_x_ henceforth.^[^ ^40^ ^]^ The following are some potential reactions that could take place during preparation:^[^ ^38^, ^39^ ^]^

(1) $$Ti_{3}\mathrm{Al}C_{2}\left(s\right)+3\mathrm{HF}\left(\mathrm{aq}\right)\to Ti_{3}C_{2}\left(s\right)+\mathrm{Al}F_{3}\left(\mathrm{aq}\right)+3/2H_{2}\left(g\right)$$

(2) $$Ti_{3}C_{2}\left(s\right)+2\mathrm{HF}\left(\mathrm{aq}\right)\to Ti_{3}C_{2}F_{2}\left(s\right)+H_{2}\left(g\right)$$

(3) $$Ti_{3}C_{2}\left(s\right)+2H_{2}O\left(\mathrm{aq}\right)\to Ti_{3}C_{2}{\left(\mathrm{OH}\right)}_{2}\left(s\right)+H_{2}\left(g\right)$$

(4) $$Ti_{3}C_{2}{\left(\mathrm{OH}\right)}_{2}\left(s\right)\to Ti_{3}C_{2}O_{2}\left(s\right)+H_{2}\left(g\right)$$

In this general etching method, we follow protection rules for HF treatment; therefore, it should be utilized in conjunction with a mixture of fluoride salt/acid (Figure 2b).^[^ ^41^ ^]^ As an alternative, it has been demonstrated that the approach of producing HF in situ by combining fluorine‐containing salts with strong acids can be used as a safer etching solution as summarized in the following chemical equation:^[^ ^38^ ^]^

(5) $$\mathrm{LiF}\left(\mathrm{aq}\right)+\mathrm{HCL}\left(\mathrm{aq}\right)\to \mathrm{HF}\left(\mathrm{aq}\right)+\mathrm{LiCl}\left(\mathrm{aq}\right)$$

wherein, ammonium bifluoride (NH_4_HF_2_) has been used as an etchant for Al from Ti_3_AlC_2_ in water:^[^ ^39^ ^]^

(6) $$\begin{array}{ccc} Ti_{3}\mathrm{Al}C_{2}\left(s\right) & + & 3NH_{4}HF_{2}\left(\mathrm{aq}\right)\to Ti_{3}C_{2}\left(s\right)\\ & + & \;{\left(NH_{4}\right)}_{3}\mathrm{Al}F_{6}\left(\mathrm{aq}\right)+3/2H_{2}\left(g\right) \end{array}$$

(7) $$\begin{array}{c} Ti_{3}C_{2}\left(s\right)+\mathrm{aN}H_{4}HF_{2}\left(\mathrm{aq}\right)+bH_{2}O\left(\mathrm{aq}\right)\\ \to {\left(NH_{3}\right)}_{c}{\left(NH_{4}\right)}_{d}Ti_{3}C_{2}+{\left(\mathrm{OH}\right)}_{x}F_{y}\left(s\right) \end{array}$$

Comparing HF‐based etching and mild etching, acid‐ or salt‐based HF etching requires a longer time for etching. The advantages include an enhanced open nanostructure with uniform growth, reduced defects, longer flakes, a large number of termination groups, and excellent electrical conductivity.^[^ ^42^ ^]^ Chemical etching techniques based on fluoride, which are the foundational MXene synthesis processes, introduce innovative F‐free synthesis (Figure 2c,d), which opens a new path for MXene preparation.^[^ ^42^, ^43^ ^]^ Wherein, several safely handled etching salts have been used for alkali etching. This etching process obtains excellent quality and oxygen and −OH terminated MXene by using electrodeposition and hydrothermal techniques in HCl, NaOH, and KOH solutions.

The temperature and concentration of HCl, NaOH, and KOH are important parameters in the reaction: at lower temperatures, the oxidation of Ti and by‐products such as TiO_2_ become drawbacks. Recently, several innovative methods have been presented for the MXene synthesis process. These include photolithography, where UV irradiation on the MAX phase in the presence of acid results in the production of an MXene phase with –OH and –Cl surface‐terminated groups.^[^ ^44^ ^]^ In, preparation of MXene using an iodine‐assisted etching method was reported in which non‐aqueous iodine (I_2_) was used^[^ ^45^ ^]^ (Figure 2e). During the etching process, a solution containing anhydrous acetonitrile was optimized at a heating temperature of 100 °C. The obtained MXene layers had a thickness of a few nm. This synthesis approach was simple, toxic chemical‐free, and inexpensive compared to other synthesis methods. This scalable approach produced a higher yield of MXene product than other MXene etching methods. Also, an ion‐induced MXene etching method was introduced^[^ ^46^ ^]^ (Figure 2f). This method includes various successive processes such as reactions, washing, and centrifugation. This method is exceptional due to the large nanosheets produced and the absence of re‐stacking issues. Later, other strategies for MXene synthesis via a scalable approach to the industrial level were explored (Figure 2g), involving a large 1L MXene reactor in a 3D structure model system with a cooling tank system and interior jacket for temperature control during the chemical reaction.^[^ ^47^ ^]^ Another method for MXene synthesis is the bottom‐up approach, where fluorine‐containing solutions have been used. This method is highly corrosive and the final yield of MXene was lower, but the product exhibited high crystallinity. Generally, bottom‐up approach methods include gas‐phase and chemical vapor deposition methods.^[^ ^48^ ^]^ In this synthesis process, a TiCl_3_ precursor was heated to 770 °C and reacted successively with CH_4_, resulting in the formation of MXene (Figure 2h). To date, various methods have been described for the preparation of MXene for useful applications via different synthetic protocols. In summary, these etching techniques hold promise for enhancing the quality of MXene processing by modifying the surface functional groups of MXene. MXenes synthesized using fluorine‐based etching show surface termination groups such as –OH, O, and F. MXenes produced through alkali‐ and fluorine‐free etching methods exhibit fewer F‐terminated MXenes, with similar results reported in the case of molten salt etching methods.^[^ ^49^ ^]^ Oxygen termination groups are essential for energy storage applications such as supercapacitors.^[^ ^50^ ^]^ The oxygen‐terminated surface groups of MXenes offer more cycle stability and electrochemical performance. Additionally, the oxygen termination group provides excellent interactions with electrolyte ions for rapid charge‐storage processes. Compared to HF‐based etching, the oxygen termination group using mild etching processes delivers greater advantages, including lower defects, increased flake size, more active sites, high surface area, and better electrical conductivity.^[^ ^24^, ^33^, ^51^ ^]^ Thus, the surface oxygen termination group is more essential for supercapacitor applications.

MXene can exhibit narrow band‐gap semiconductor properties by altering its functional termination groups. F and OH groups exert comparable effects on the electrical structure of MXene due to oxidation states, facilitating electron acceptance.^[^ ^52^ ^]^ Furthermore, based on their respective electrical conductivity, MXenes can be classed as metallic, semi‐metallic, or semiconducting. Experimentally, it was found that MXene has excellent conductivity due to its morphology and chemical composition. For example, MXene exhibits a high conductivity of 9880 S cm^−1^. This superior electronic conductivity facilitates faster charge transport and stability for supercapacitors.^[^ ^53^ ^]^ Based on hypothetical scheming, bare MXene exhibits a strength of 92–161 N m^−1^ and a stretch and deform property of 0.33 TPa, indicating greater mechanical stability,^[^ ^54^, ^55^ ^]^ which is helpful for flexible supercapacitor devices. Due to its easy oxidation, MXene can oxidize at room temperature over 15 days.^[^ ^56^ ^]^ Thus, MXene is stored in the dark at 5 °C in Ar‐filled containers to prevent oxidation from light and air.^[^ ^57^ ^]^ MXene is not oxidized below 800 °C in the Ar atmosphere.^[^ ^58^ ^]^ It should also be noted that the preparation of MXene involves the use of various solvents, which has a crucial impact on electrochemical performance.^[^ ^59^ ^]^ The versatile MXene properties discussed above are applied in numerous fields such as energy storage, batteries, supercapacitors, photocatalysis, water purification, sensors, electronics, and electrocatalysis.^[^ ^6^, ^8^, ^18^, ^35^, ^50^, ^52^, ^60^ ^]^ Due to the effective electronic, mechanical, and electrochemical properties of 2D MXene, it has been highly reported in the literature focusing on supercapacitors.

## 3 MXene Supercapacitor Properties

**Figure 3:**
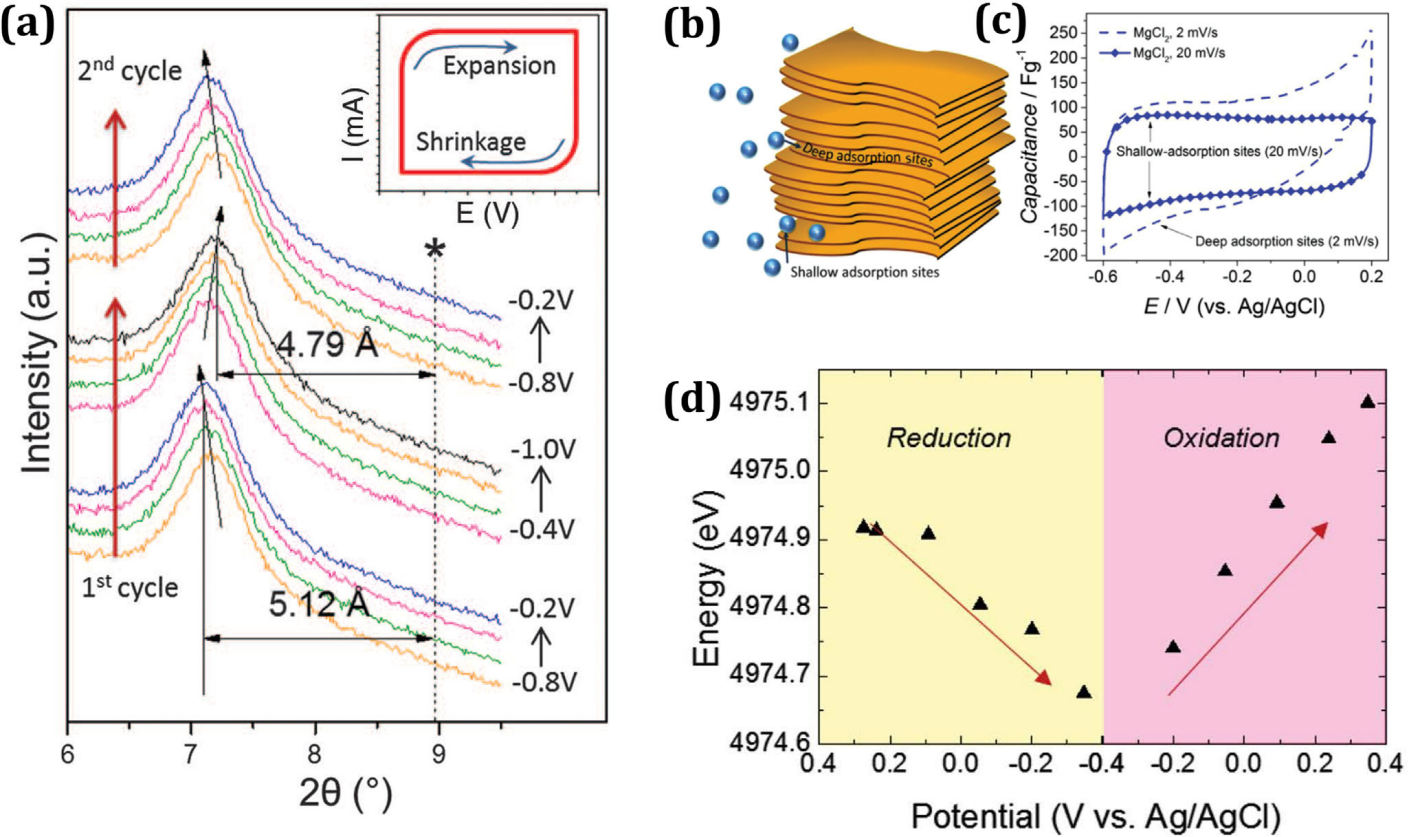
In situ XRD analysis for MXene intercalation in the potential range of −1 to −0.2 V in 1 M KOH electrolyte, wherein a) vertical lines indicate the initial peak position (0002) of the MXene electrodes before cell assembly while inclined vertical arrows demonstrate the shift in (0002) peak position. Insets explain the expansion and shrinking of c lattice parameters during cycling. Reproduced with permission.^[^ ^66^ ^]^ Copyright 2013, The American Association for the Advancement of Science. Intercalation of electrolyte ion on exfoliated and multi‐layered nanosheet of MXenes presented in b) by an illustration of MXene multilayer covering shallow and deep adsorption sites, which is closer to gap opening and interior part. c) Capacitance of MXene/carbon measured at scan rates of 2 and 20 mV s^−1^ in 1 M MgCl_2_ electrolyte, corresponding to deep and shallow adsorption sites. Reproduced with permission.^[^ ^68^ ^]^ Copyright 2015, John Wiley and Sons. d) Electrochemical in situ X‐ray data of the Ti edge energy versus potential show that a potential sweep between 0.275 and −0.35 V shifts the Ti edge to lower energy, indicating a reduction in its average oxidation state. Reproduced with permission.^[^ ^69^ ^]^ Copyright 2015, John Wiley and Sons.

MXene has been regarded as a potentially useful supercapacitor^[^ ^61^, ^62^, ^63^, ^64^ ^]^ and has been widely employed within a negative potential window.^[^ ^65^ ^]^ This is attributed to its variable valency, which induces charge transfer facilitated by transition metals, its layered structure with open channels facilitating ion transportation, and its high conductivity for electron transport. Consequently, MXene has been categorized as a negative electrode material with pseudo‐capacitance as shown in **Figure** **3**a. MXene involves charge storage mechanisms (due to reversible intercalation of Li^+^, Na^+^, NH_4_^+^, etc.) over its surface.^[^ ^66^ ^]^ During cycling, the ion adsorption and desorption processes result in interlayer space expansion in MXene. The modifications of stacked MXene nanosheets during adsorption or desorption processes, whether undergoing contraction or expansion, have been elucidated through various analytical techniques, including in situ XRD, atomic force microscopy, and electrochemical quartz‐crystal admittance.^[^ ^67^ ^]^ Larger‐sized cations enlarge the stacked layer structures of MXene, which suggests that there is cation intercalation between the MXene and cations. Shallow‐adsorption sites exist near the edges of the MXene nanosheet, whereas deep‐adsorption sites for ion adsorption exist in the interior part of the stacked MXene nanosheet (see Figure 3b). As shown in Figure 3c, it is reported that at a reduced scan rate, electrolyte ions were moved deep inside the MXene electrode, resulting in increased capacitance.^[^ ^68^ ^]^ To develop deeper into the reaction kinetics of MXene in the electrochemical process, an *in‐situ* analysis carried out in 1 M H_2_SO_4_ found that MXene electrode changed oxidation states concerning the applied potential window (see Figure 3d).^[^ ^69^ ^]^

Here, it should be noted that because MXene operates exceptionally well electrochemically at negative potentials, an irreversible oxidation process may be reflected. MXene has the following interesting features: higher conductivity for super‐fast hopping electrons; an open porous nanostructure for superior adsorption and desorption processes; TMO/TMD/LDH to allow electrolyte ions transmission; and higher oxygen functional groups and redox active sites, which reflect its pseudo‐capacitive performance.^[^ ^70^, ^71^ ^]^ There are several reports available on MXene used for supercapacitors, including an example where MXene exhibited an aerial capacitance of 579 mF cm^−2^.^[^ ^72^, ^73^ ^]^ The electrochemical performance of MXene can be improved by doping to expand the stacking of the nanosheets.^[^ ^74^ ^]^

For example, p‐doped MXene for electrochemical energy storage delivers a maximum capacitance of 31.11 mA h g^−1^ (1 A g^−1^; 1 M KOH).^[^ ^75^ ^]^ The authors here demonstrated that doping with phosphorus leads to an expansion of the interlayer spacing of MXene nanosheets. When electrochemical testing with seawater as an electrolyte, the MXene electrode supercapacitor showed volumetric specific capacitance of 67.7 F g^−1^ at a current density of 1 A g^−1^ and 96.6% retention over 5000 cycles.^[^ ^76^ ^]^ The obtained electrochemical performance of the MXene electrode is similar to the aqueous KOH electrode, suggesting the importance of seawater as an electrolyte. In summary, the highly stacked 2D nanosheet‐like nanoarchitectures of MXene surfaces offer improved electrochemical performance, potentially enabling the synthesis of supercapacitors with high energy/power densities and exceptional stability.

## 4 Synthesis Methods for MXene and 2D Transition Metal Oxide Nanocomposites

**Figure 4:**
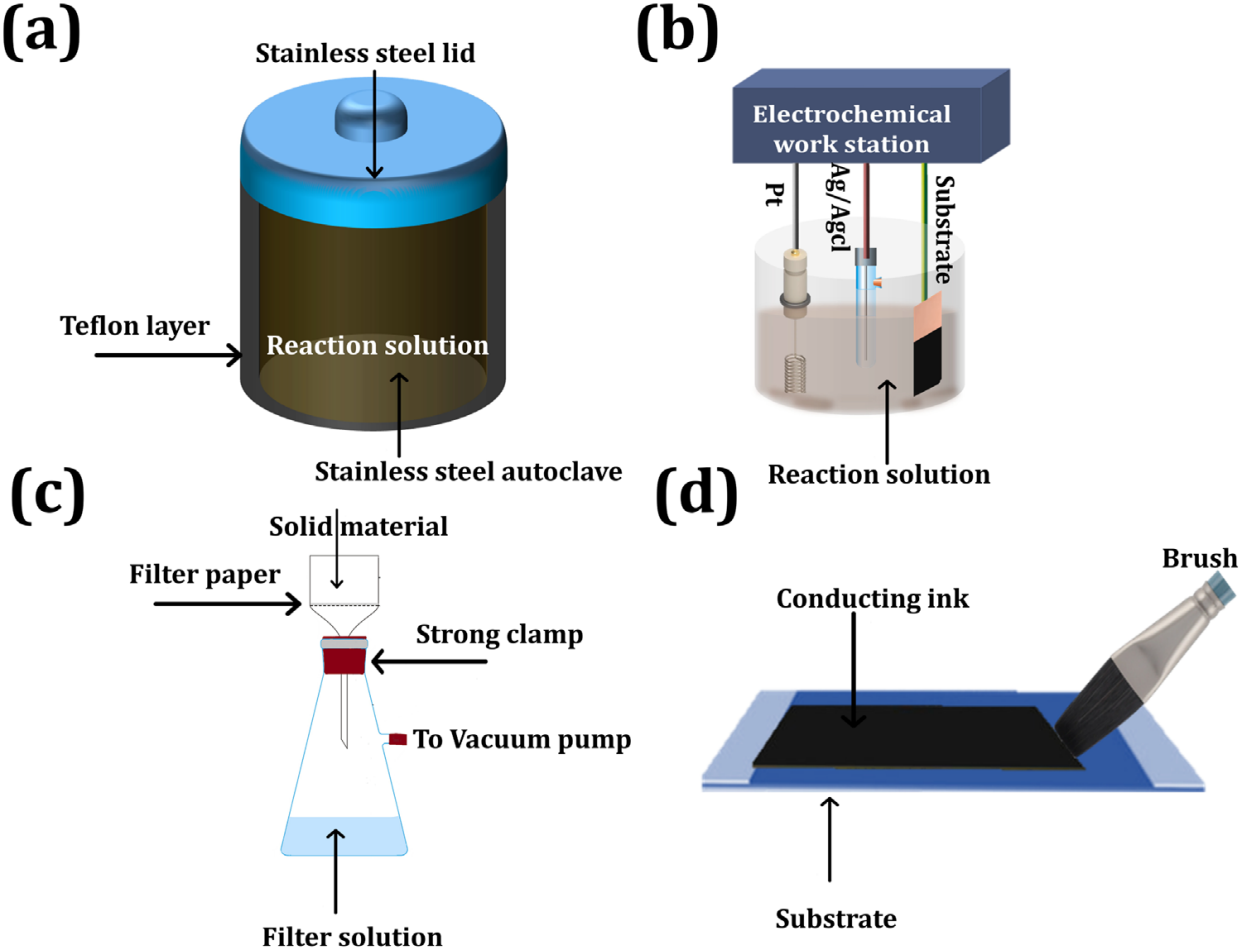
Different synthesis methods for MXene‐based nanocomposites: a) Schematic of Teflon‐lined stainless‐steel autoclave for hydrothermal method (Re‐draw from reference paper).^[^ ^82^ ^]^ b) Representative configuration of an electrodeposition method with Pt: counter; Ag/AgCl: reference and substrate: working electrode (Re‐draw from reference paper).^[^ ^83^, ^84^ ^]^ c) Schematic of vacuum‐assisted filtration method for synthesis of solid material in the presence of an external vacuum pump.^[^ ^87^ ^]^ d) Schematic of brush painting method using conducting ink (Re‐draw from reference paper).^[^ ^90^ ^]^

Various chemical methods have been used to prepare MXene‐based nanocomposites such as hydrothermal, electrodeposition, vacuum filtration, coating methods, etc.^[^ ^77^, ^78^ ^]^ The electrochemical properties of the prepared 2D MXene and other 2D material nanocomposites vary with the synthesis approach as each method involves optimized parameters such as reaction solution pH, chemical composition, temperature, and others.^[^ ^79^, ^80^ ^]^ Overall, the hydrothermal method is the most commonly used technique for synthesizing MXene‐based nanocomposites as illustrated in **Figure** **4**a. In this method, when the reaction solution is heated to its maximum temperature and reaches critical vapor pressure, a precipitate forms. The hydrothermal method produces highly crystalline nanostructures with varying sizes and shapes.^[^ ^81^, ^82^ ^]^ In electrochemical deposition, MXene nanocomposite was deposited onto a conducting substrate (carbon cloth, nickel foam, and copper and stainless‐steel substrate)^[^ ^83^ ^]^ (Figure 4b). In the electrodeposition method, electrochemical redox reactions occur in a metal salt solution. At a later stage, when an electric field is applied to the substrate relative to the reference electrode, the metal is uniformly coated onto the conducting substrate.^[^ ^84^ ^]^ Electrodeposition offers several advantages, such as the controlled growth of nanostructures by adjusting the solution pH, current, and voltage range as well as the uniform deposition of MXene nanocomposites on conducting substrates of various sizes and shapes.^[^ ^85^ ^]^ Vacuum filtration is a simple and common method for preparing MXene‐based nanocomposites in which 2D MXene film is obtained via filter through a well‐dispersed solution containing MXene and metal oxide (Figure 4c). After total filtration of the reaction solution, MXene nanocomposite film was obtained over the filter membrane.^[^ ^86^ ^]^ The thickness of the prepared MXene nanocomposite layer was controlled by adjusting the vacuum pressure and the chemical composition of the reaction solution.^[^ ^87^, ^88^ ^]^ Several non‐conventional synthesis methods, such as dip‐coating and painting, have been introduced for the synthesis of MXene nanocomposites. These approaches are well‐organized, simple, and cost‐effective. In this method, MXene nanocomposite inks have been used for patterning different architectures for flexible electronics and energy storage devices (see Figure 4d).^[^ ^89^, ^90^ ^]^

The following section explains the various methods used for the synthesis of MXene‐based nanocomposites and related supercapacitor properties. The enhanced electrochemical performance of MXene‐based nanocomposite exposes higher capacitance and cycling stability than that of pristine MXene.

### 4.1 MXene and 2D Transition Metal Oxide Composites

Nanocomposite MXene/TMOs are auspicious supercapacitor electrodes due to their higher energy and power density, and longer stability.^[^ ^91^ ^]^ In MXene/TMO nanocomposites, TMOs avoid the rearrangement and aggregation of MXene sheets, and transport ions due to their higher conductivity. Furthermore, TMOs might not only separate the MXene stacked layer but also improve interfacial contact and improve the supercapacitor performance of MXene and TMO‐based nanocomposites. MXene‐based nanosheets play an essential role as current collectors and support different sized and shaped TMOs.^[^ ^92^ ^]^ In short, the MXene/TMO nanocomposite is a promising energy storage device due to the synergistic interaction between the high‐capacity TMO and conducting MXene.^[^ ^93^, ^94^ ^]^ 2D TMOs include, for example, MnO_2_, MoO_4_, RuO_2_, MoO_3_, Co_3_O_4_, NiO, WO_3_, and Fe_2_O_3_.^[^ ^95^, ^96^, ^97^ ^]^ Many MXene/TMO nanocomposite materials have been created to date, combining the benefits of MXene and TMO materials. Manganese oxides (MnO_2_) are among the most studied oxides when paired with MXene due to their higher capacitance value, non‐poisonous nature, reasonable price, and easy synthesis.^[^ ^98^, ^99^ ^]^ In addition, MnO_2_ metal oxide showed oxidation states from Mn^3+^ to Mn^4+^ and capacitance of 1370 F g^−1^.^[^ ^95^ ^]^ Consequently, extensive research has been done on MnO_2_ and its MXene nanocomposite to create high‐performance supercapacitors.^[^ ^100^, ^101^, ^102^, ^103^, ^104^ ^]^ For example, MXene/MnO_2_ nanowire has been fabricated by solution processing. MnO_2_ nanowires are loaded across the surface of MXene, resulting in the development of a continuous conducting network for electron transport. The MnO_2_/MXene electrode exhibited boosted supercapacitor performance with capacitance of 205 mF cm^−2^ and stability ≈98% over 10000 cycles.^[^ ^105^ ^]^ Another way is to prepare MnO_2_/MXene nanocomposites is via a chemical route.^[^ ^106^ ^]^ The electrochemical performance of MnO_2_/MXene showed a capacitance of 130 F g^−1^. The flexible supercapacitor device showed power density of 0.7 mW h cm^−2^ (80.0 mW cm^−2^). Fabrication of MnO_2_ and MXene using hydrothermal method leads to porous structures.^[^ ^107^ ^]^ MnO_2_/MXene nanocomposite has a large surface area due to its wide pore size (ranging 5–9 nm) and, as a result, the nanocomposite performs well as a supercapacitor, with capacitance of 181 F g^−1^ and retention of 91% (5000 cycles).

**Figure 5:**
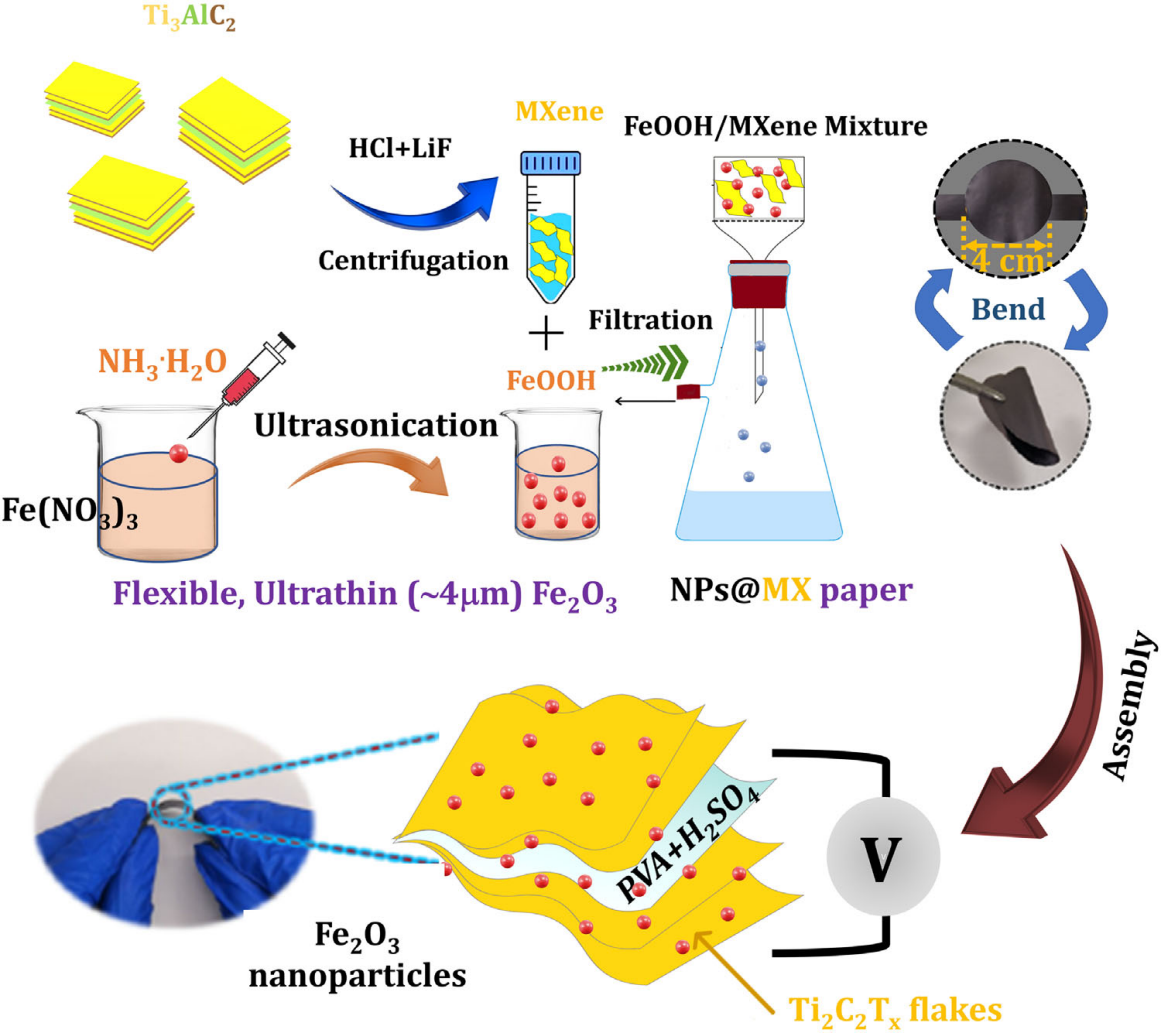
Synthesis of MXene and transition metal oxide composites‐based nanocomposite with an example preparation of MXene/Fe_2_O_3_ using vacuum filtration (Re‐draw from reference paper).^[^ ^109^ ^]^ in which a solution containing MXene and FeOOH suspension was vacuum‐filtered through cellulose paper.

Fe_2_O_3_ is a well‐known TMO‐based pseudo‐capacitive electrode material, having a maximum capacitance of ≈3625 F g^−1^.^[^ ^108^ ^]^ Utilizing these characteristics, Fe_2_O_3_ nanoparticles were decorated over the MXene surface using a self‐assembly process^[^ ^109^ ^]^ as shown in **Figure** **5**. The Fe_2_O_3_ nanoparticles built over the MXene surface provided a greater inter‐gap extension during adsorption/desorption processes, thereby providing more reactive sites and improving supercapacitor performance. Electrochemical analysis shows that Fe_2_O_3_ nanoparticles at the MXene electrode exhibit capacitance of 584 F g^−1^. The supercapacitor device obtained capacitance of 920 F cm^−3^ with 95% retention (10000 cycles), an energy, and power density of 29 W h L^−1^ and 213 W L^−1^, respectively. In this article, cellulose paper acts as a free‐standing current collector following the vacuum filtration of a solution containing MXene and FeOOH suspension. During the vacuum filtration process, MXene nanosheets and FeOOH were electrostatically attracted to each other. As a result, conducting metallic networks formed over the cellulose paper as shown in Figure 5. The vacuum filtration synthesis of MXene/FeOOH nanocomposite is simple, fast‐coating, and convenient for enlarging the spacing of MXene nanoflakes. Also, with incorporation of a secondary TMO electrode over the MXene nanoflakes enhances the electrochemical performance of more active sites. Based on the excellent results obtained from synthesis and electrochemical analysis, it is feasible to synthesize large‐area MXene nanocomposites using vacuum filtration with minimal chemical waste and time consumption (Figure 5). The synthesized *α*‐Fe_2_O_3_C/MXene via a hydrothermal route realized specific capacitance of ≈40 F g^−1^ with stability of 84%.^[^ ^110^ ^]^ Another report described MXene/*α*‐Fe_2_O_3_ synthesis using a self‐assembly method. SEM results showed that MXene/*α*‐Fe_2_O_3_ reflected *α*‐Fe_2_O_3_ nanoparticles and MXene nanosheets. These nanocomposites’ highest gravimetric specific capacitance was 289 F g^−1^ with stability of 97% over 2000 cycles.^[^ ^111^ ^]^

Because cobalt oxide (CoO_x_) is inexpensive and has strong redox characteristics, it is being investigated extensively as a supercapacitor electrode.^[^ ^112^ ^]^ The electrochemical testing of Co‐MXene displayed capacitance of 1081 F g^−1^. The asymmetric supercapacitor device showed high an energy and power densities of 26 W h kg^−1^ and 1400 W kg^−1^, respectively.^[^ ^113^ ^]^ Similarly, another report describes the use of MXene and cobalt oxide nanocomposite for supercapacitor applications. SEM images revealed that the cobalt nanoparticles were well settled over the MXene nanosheets, which enhances the charge transfer rate. In a half‐cell, the optimized Co@MXene electrode obtained higher capacitance of 732 F g^−1^ and stability of 83% (5000 cycles). The asymmetric device (Co/MXene‐2//activated carbon) demonstrated maximum an energy density, and power density of 26 W h kg^−1^ and 700 W kg^−1^, respectively.^[^ ^114^ ^]^ In both the research articles above, cobalt oxide nanoparticles were coated over MXene nanosheets with controlled and limited growth, allowing improved interface charge transportation.

The MoO_3_ nanobelts and MXene electrode were prepared using the vacuum‐assisted method. The capacitance of the MoO_3_/MXene electrode was 545 F g^−1^. SEM images show that MoO_3_/MXene, as nanobelts inserted into MXene, not only helps expose additional surface area with active sites for rapid electron transport, but by using the MoO_3_ nanobelts its thickness increases significantly and, thus, its volumetric performance. Furthermore, the assembled symmetric supercapacitor device exhibited an energy, and power density of 44 W h L^−1^ and 1782 W L^−1^, respectively.^[^ ^115^ ^]^ MXene‐MoO_2_ electrodes were prepared using the hydrothermal method. MXene nanosheets were discovered to have formed into a homogeneous film with noticeable creases.^[^ ^116^ ^]^ MXene‐MoO_2_ film had fewer creases and was rougher than pure MXene film. This could be because the MoO_2_ was dispersed over the MXene layer. The MXene‐MoO_2_ film showed areal capacitance of ≈19 mF cm^−2^. Another strategy employed MoO_3_ nanorods on the MXene surface.^[^ ^117^ ^]^ BET analysis estimated that the specific surface area of D‐MXene/MoO_3_@IL was 36 m^2^ g^−1^ with the capacitance of 1680 F g^−1^. MXene nanosheets with MoO_3_ represent a unique nanostructure that is more suitable for supercapacitors due to the maximized surface area and increased stacked spacing within the MXene nanosheets, which improves electrolyte–nanocomposite interaction.

WO_3_ with MXene was produced using the hydrothermal method.^[^ ^118^ ^]^ FE‐SEM images of the mono‐WO_3_–MXene hybrid show WO_3_ nanorods distributed over the surface of MXene. The synthesis of Hexa WO_3_ showed a characteristic length of 5 nm. The SAED pattern is reflected in the crystalline nature of the mono‐WO_3_ nanorods. Electrochemical analysis showed specific capacitance of mono‐WO_3_, exhibiting the highest capacitance for Hexa WO_3_–MXene and excellent endurance (92%) over 5000 cycles. In this research, MXene serves as a basis for developing WO_3_, which improves the interfacial contact between both electrodes, ultimately increasing the electrolyte transfer rate. W_18_O_49_/MXene nanocomposite was synthesized using the hydrothermal method.^[^ ^119^ ^]^ Electrochemical analysis found that the specific surface area for the W_18_O_49_/MXene electrode was 39 m^2^ g^−1^. The W_18_O_49_/MXene exhibited a higher supercapacitor performance of 696 F g^−1^, which was enhanced as compared to the pristine sample, with a retention of 85% (10000 cycles). A supercapacitor device obtained a capacitance of 101 F g^−1^ (1 A g^−1^), an energy density of 10 W h kg^−1^, and power density of 18000 W kg^−1^. The W_18_O_49_ nanoflowers were covered with MXene nanosheets both on the outside and inside, resulting in substantial synergistic effects between the electrodes. In addition, this synthesis approach creates a fast‐track for transporting electrolyte ions with minimal resistance.

RuO_2_/MXene nanocomposite was synthesized using a chemical solution method.^[^ ^120^ ^]^ Three electrochemical analyses showed RuO_2_/MXene exhibited higher capacitance of 612 F g^−1^ (2 A g^−1^: electrolyte H_2_SO_4_) and 97% retention (10 000 cycles). The obtained nanocomposite MXene/TMOs nanocomposite demonstrated a highly nonporous nanostructure. The synthesis of MXene and RuO_2_ on carbon cloths via drop casting and the hydrothermal method^[^ ^121^ ^]^ represents a notable advancement. Notably, MXene proves particularly adept at generating RuO_2_ nanoparticles without aggregation. This synthesis yields RuO_2_ nanoparticles abundant in active reaction sites and facilitates rapid charge transfer, enhancing electrochemical performance significantly. The asymmetric devices achieved an energy, and power densities of 37 µW h cm^−2^ and 40 mW cm^−2^, respectively, and retained 86% over 20000 cycles. It was observed that 2D TMO nanostructures were assembled over the MXene nanosheets, which efficiently not only protect from oxidation but also from re‐stacking of MXene. This increases the accessibility of electrolyte ions, extends the potential window, and enlarges the active surface area of the MXene/TMO nanocomposites to provide conductive routes for charge adsorption–desorption. The result is increased capacitance and retention over longer cycles. **Table** **1** shows the performance of different 2D MXene and 2D TMO materials, and compares the electrochemical data of 2D/2D MXene/TMOs, showing that MXene and TMO nanocomposites have remarkable supercapacitor performance.

**Table 1:** Electrochemical performance of MXene/TMO‐based supercapacitors.

Sr. No.	Material	Synthesis method	Specific capacitance	Stability/Cycles	Refs.
1	MXene/MnO_2_	Solution processing	205 F cm^−3^	98%/10000	[105]
2	MXene/MnO_2_	Refluxing	130 F g^−1^	80%/10000	[106]
3	MnO_2_/MXene/CNT	Hydrothermal	181 F g^−1^	91%/5000	[107]
4	MXene/Fe_2_O_3_	Vacuum‐filtered	584 F cm^−3^	121%/13000	[109]
5	MXene/*α*‐Fe_2_O_3_/C	Hydrothermal	40 F g^−1^	84%/——–	[110]
6	MXene/*α*‐Fe_2_O_3_	Electrostatic	289 F g^−1^	97%/2000	[111]
7	MXene/Co_3_O_4_	Hydrothermal	1081 F g^−1^	——–	[113]
8	MXene/Co	Hydrothermal	732 F g^−1^	83%/5000	[114]
9	MXene/MoO_3_	Vacuum filtration	545 F g^−1^	100%/5000	[115]
10	MXene/MoO_2_	Hydrothermal	19 mF cm^−2^	————–	[116]
11	MXene/MoO_3_	Solvothermal	1680 F g^−1^	70%/1000	[117]
12	MXene/WO_3_	Hydrothermal	566 F g^−1^	92%/10000	[118]
13	MXene/W_18_O_49_	Hydrothermal	696 F g^−1^	85%/10000	[119]
14	MXene/RuO_2_	Chemical solution	612 F g^−1^	97%/10000	[120]
15	MXene/RuO_2_	Hydrothermal	388 F g^−1^	88%/20000	[121]

### 4.2 MXene and 2D Transition Metal Dichalcogenide Composites

MXene‐TMD nanostructures have recently been documented for use in supercapacitors. The synergetic effect the MXene‐TMD nanostructures introduces a new area of research in which the problem of nanosheet stacking has been successfully overcome. Thus, maintaining the interlayer stacked nanosheets with wide open spaces (10 Å) facilitates rapid electrolyte ion transfer between the MXene‐TMD composites with tunable nanostructures, making them promising electrode materials for supercapacitors in energy storage applications. The most recent advancements in TMD/MXene nanocomposite nanostructures, organized in the following sections, represent outstanding materials for supercapacitors. Their electrochemical performance surpasses and stands on par with that of other electrode materials such as TMO, LDH, and carbon.^[^ ^122^ ^]^ On the other hand, TMDs’ important drawbacks are a narrow voltage window, structural disintegration, and poor cycling stability.^[^ ^123^, ^124^ ^]^

2D MXene/2D TMD nanocomposites were produced and published. Cobalt sulfide (CoS) offers several advantages, including strong electrical conductivity and specific capacity as well as an abundance of electrochemical redox sites. Combining CoS with MXene enhances cycling performance. Hence, CoS was combined with MXene flakes via the hydrothermal method.^[^ ^125^ ^]^ From FE‐SEM images, MXene/CoS nanostructures are observed coated on nickel foam. In cyclic voltammetry curves of the MXene/CoS electrode, the oxidation peak is recorded near 0.5 and 0.3 V, which indicates battery‐type performance. The capacitance of MXene/CoS electrodes is 447 F g^−1^. The fabricated MXene‐CoS//activated carbon supercapacitor device delivered capacitance of 190 mA h g^−1^ and 93% stability over 3000 cycles. The CoS_2_ nanoparticles are directly designed on MXene surfaces by the solvothermal method.^[^ ^126^ ^]^ From SEM images of the MXene/CoS_2_ nanocomposites, it was clearly observed that there are two morphological types: spherical and lamellar. The synthesized MXene/CoS_2_ nanocomposite delivered specific capacitance 1320 F g^−1^ and exhibited stability of 78% over 3000 cycles. Moreover, the full‐cell device has a wide potential window of 1.6 V and obtained an energy density (power density) as high as 28.8 W h kg^−1^ (800 W kg^−1^), and retained 98% at 5000 scan cycles. CoS nanowires and MXene were synthesized using the solvothermal method.^[^ ^127^ ^]^ BET analysis showed that surface areas of CoS nanowires and MXene are ≈20 m^2^ g^−1^ and 21 m^2^ g^−1^, respectively, which is relatively lower than that of CoS/MXene nanocomposite. Hence, the prepared CoS/MXene showed capacitance of 528 F g^−1^ with 99% stability (20 000 cycles), which can be attributed to the interaction between the MXene and CoS nanocomposite. The CoS nanowires are wrapped around entire MXene nanosheets, forming a conductive network that is expected to boost electrochemical performance and buffer volume expansion.

**Figure 6:**
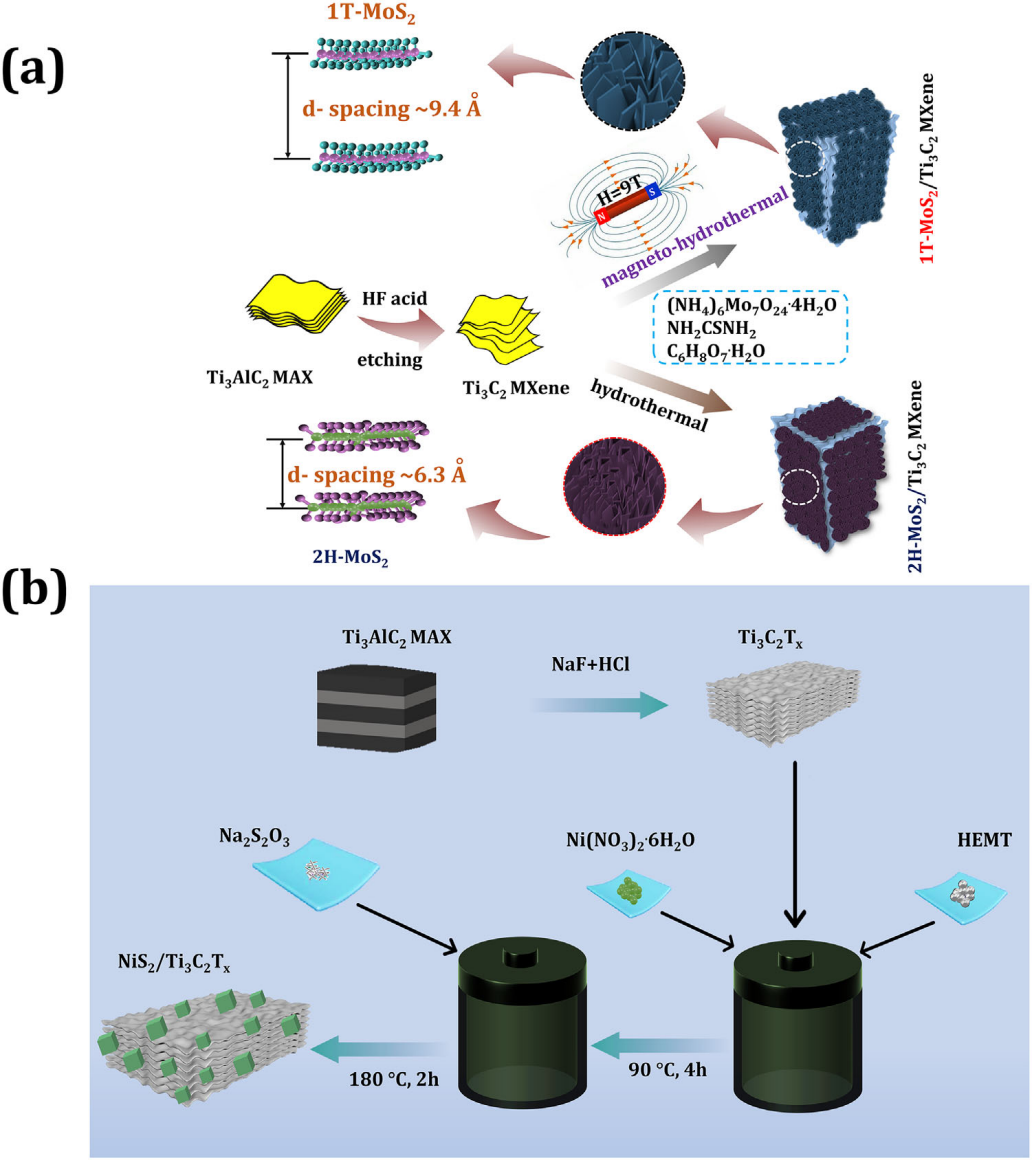
Synthesis of MXene and transition metal dichalcogenide‐based nanocomposite using hydrothermal methods. a) Illustration of synthesis for MoS_2_/MXene nanocomposite using magneto‐hydrothermal synthesis under an applied magnetic field of 9T, resulting in an MXene nanosheet covered by a MoS_2_ nanosheet (Re‐draw from reference paper).^[^ ^129^ ^]^ b) Schematic of synthesis for NiS_2_/MXene composite using hydrothermal method. After the hydrothermal process, randomly distributed nanocube‐like NiS_2_ nanostructures assembled over the MXene surface (Re‐draw from reference paper).^[^ ^136^ ^]^

The CoSe_2_/MXene electrode was 3D‐printed for supercapacitor applications.^[^ ^128^ ^]^ Here, polyhedron CoSe_2_ with an average particle size of 200 nm was coated on the MXene layer. TEM analysis revealed a reflected inter‐planer distance of ≈0.26 nm, which belongs to the (210) plane of CoSe_2_. Electrochemical analysis confirmed that the CoSe_2_/MXene electrode obtained 540 mA h g^−1^. MoS_2_, as one of the characteristic 2D TMDs, has been used for the synthesis of MXene‐based nanocomposite due to its high capacitance of ≈210 Fg^−1^. MoS_2_/MXene nanocomposites were synthesized using magneto‐hydrothermal synthesis.^[^ ^129^ ^]^ SEM images of the MoS_2_ electrode showed nanosheets and graphene‐like morphology. The 1T‐MoS_2_/MXene electrode exhibited a higher specific capacitance of 386 F g^−1^ (at 1 A g^−1^). At the end, the 1T‐MoS_2_/MXene‐based supercapacitor device showed excellent capacitance of 347 mF cm^−2^ (2 mA cm^−2^) and retention of ≈91% at 20000 cycles. In summary, this work presents a unique scientific approach to MXene nanocomposite synthesis as illustrated in **Figure** **6**a. This innovative concept involves using a magnetic field‐assisted hydrothermal process to consistently coat 2H‐MoS₂ nanosheets on MXene rather than relying on conventional hydrothermal methods. Interestingly, the presence of a magnetic field during the hydrothermal process led to the formation of 1T‐MoS₂/MXene, with a wide interlayer spacing of ≈9.4 Å. The resulting 1T‐MoS₂ nanosheets had a thickness of ≈6.5 nm and exhibited high stability. A key feature of the 1T MoS₂/ MXene electrodes is their open interlayer spacing, which not only facilitates rapid electron transport but also enhances the diffusion of electrolytes into the interior. In summary, the 1T‐MoS₂/MXene nanocomposite synthesized via magneto‐hydrothermal methods exhibits excellent electrochemical properties.

A novel C/MoS_2_/MXene electrode was prepared using a physical method. A 3D C/MoS_2_ nanoflower was introduced among 2D MXene layers using the hydrothermal method.^[^ ^130^ ^]^ SEM images of the C/MoS_2_/MXene electrode clearly show C/MoS_2_ nanoflowers and MXene nanosheets. Electrochemical study revealed that the C/MoS_2_@MXene electrode obtained capacitance of 410 F g^−1^ compared to C/MoS_2_ and MXene. MXene/MoS_2_ has greater capacitance because the prepared nanocomposite has more active sites and preserves re‐stacking in MXene and MoS_2_ while increasing the interlayer distance of MXene's nanosheets. A 1T‐MoS_2_/MXene electrode was prepared through hydrothermal synthesis.^[^ ^131^ ^]^ SEM and TEM results of the 2H‐MoS_2_ and 1T‐MoS_2_ electrodes indicate that prepared samples consisted of a nanosheet‐likenanostructure. HR‐TEM images showed that interlayer spacing was between 6 and 9 A°, related to the (002) peaks of 2H‐MoS_2_ and 1T‐MoS_2_. Electrochemical data analysis of 1T‐MoS_2_ revealed capacitance of 206 F g^−1^, which could be attributed to the enhanced conductivity of the nanosheets. 1T‐MoS_2_ combines with extremely conductive 2D MXene. In addition, the fabricated asymmetric device showed an energy (power) density of 68 mW h cm^−2^ (4500 mW cm^−2^). The MoS_2_/MXene nanocomposite was synthesized using the co‐precipitation method.^[^ ^132^ ^]^ The MoS_2_/MXene revealed a higher capacitance of 342 F g^−1^ (0.4 A g^−1^) and 99% stability over 10000 cycles. 2D MoS_2_ overcomes the self‐re‐stacking issue in MXene nanosheets via the insertion of MoS_2_ nanosheets into the 2D MXene layer.

For example, the hierarchical structure of Ni_3_S_2_/MXene composite material is synthesized using the hydrothermal method.^[^ ^133^ ^]^ An innovative research methodology was adopted in developing some 2D MXene‐2D Ni_3_S_2_. The advantage of Ni_3_S_2_ is higher theoretical capacity (≈590 mA h g^−1^), various redox state rates, and responsive interfacial features with 2D MXene materials. The SEM result found that for Ni_3_S_2_/MXene, polyhedral Ni_3_S_2_ coated on MXene had a homogeneous distribution and the edge length was ≈100 nm. Three‐electrode analysis showed that Ni_3_S_2_/MXene composite material had excellent electrochemical storage performance, stability of 98% over 5000 cycles of scanning, and obtained capacitance of 1489 F g^−1^. The electrochemical performance of the Ni_3_S_2_/MXene composite material demonstrated remarkable enhancement, attributable to the uniform coating of Ni_3_S_2_ polyhedral between the 2D nanosheets of MXene, which provided quick buffering during charging and discharging. The fabricated device showed high power density (792 W kg^−1^) and an energy density (71 W h kg^−1^) with cycle stability of 93%. The Ni_3_S_4_ nanoparticles were distributed over the MXene surface using a hydrothermal method.^[^ ^134^ ^]^ An SEM image of the prepared electrode confirmed a multi‐layered nanostructure with accordion‐like morphology, which might provide an alternative charge passage track in supercapacitors. The three‐electrode system obtained that the MXene/Ni_3_S_4_ electrode delivered capacitance of 1280 F g^−1^ at 1 A g^−1^. Furthermore, the supercapacitor device of N‐MXene/Ni_3_S_4_ delivered an excellent an energy density of 9 W h kg^−1^ at a power density of 604 W kg^−1^. NiS/MXene synthesized using inkjet printing with fabricated asymmetrical micro‐supercapacitors achieved areal capacities and volumetric capacitance of 146 mC cm^−2^ and 429 Fcm^−3^, respectively, with an energy density of 33 mW h cm^−2^.^[^ ^135^ ^]^ NiS_2_/MXene nanocomposite was prepared using a hydrothermal method as shown in Figure 6b.^[^ ^136^ ^]^ The specific capacitance and stability of MXene and NiS_2_/MXene electrodes are 15.3 and 72 mA h g^−1^, respectively, with stability of 77% over 9000 cycles. The key concept highlighted in Figure 6b of this research article is the random distribution of NiS₂ nanocubes on MXene nanoplates, which helps enhance their structural stability and electrochemical properties. In the NiS₂/MXene nanocomposite, secondary controlled growth of nickel sulfide nanostructures occurred on the MXene nanosheets, resulting in a cubic morphology as opposed to nanosheets, nanoflakes, nanorods, or nanowires. Such cube‐type NiS_2_ nanostructures present an increased surface area and average pore size of ≈4.4 m^2^ g^−1^ and 33.2 nm, respectively. As a result, there is an increased interfacial charge transportation rate between the NiS_2_ and MXene without collapsing the multilayer 2D nanosheet of MXene. Another important role of the NiS_2_ nanocube, due to its higher cube size of ≈1–2 µm, is to compensate for oxidation of the MXene nanosheet while prolonging the charge/discharge process time. NiSe_2_ was grown over MXene nanosheets and delivered high specific capacitance of 531 F g^−1^ at 1 A g^−1^.^[^ ^137^ ^]^

MXene/WS_2_ was coated on non‐conducive substrate.^[^ ^138^ ^]^ The flexible asymmetric supercapacitor exhibited capacitance of 113 F g^−1^, an energy and power density of 50 W h kg^−1^ and 813 W kg^−1^, respectively, and good cycling stability of ≈85% over 5000 cycles. **Table** **2** presents the electrochemical performance of various 2D MXene and 2D TMD materials in 2D MXene/2D TMD configurations. Notably, MXene paired with MoS_2_ and NiS_2_ nanocomposites exhibited superior or comparable performance to other TMD electrodes in the context of supercapacitor applications.

**Table 2:** Electrochemical performance of MXene/TMD‐based supercapacitors.

Sr. No.	Material	Synthesis methods	Specific capacitance	Stability/Cycles	Refs.
1	MXene/CoS	Hydrothermal	447 mAh g^−1^	95/5000	[125]
2	MXene/CoS_2_	Hydrothermal	1320 F g^−1^	78%/3000	[126]
3	MXene/CoS	Solvothermal	528 F g^−1^	99%/20000	[127]
4	MXene/CoSe_2_	3D printing	540 mAh g^−1^	——/300	[128]
5	MXene/1T‐MoS_2_	Magneto‐hydrothermal	386 F g^−1^	96%/20000	[129]
6	MXene/C/MoS_2_	Hydrothermal	410 F g^−1^	89%/5000	[130]
7	MXene/1T‐MoS_2_	Hydrothermal	206 F g^−1^	94%/10000	[131]
8	MXene/MoS_2_	Coprecipitation	342 F g^−1^	99%/10000	[132]
9	MXene/Ni_3_S_2_	Hydrothermal	1489 F g^−1^	98%/5000	[133]
10	MXene/Ni_3_S_4_	Hydrothermal	1280 F g^−1^	81%/10000	[134]
11	MXene/NiS	Ink jet printing	146 mC cm^−1^	96%/5000	[135]
12	MXene/NiS_2_	Hydrothermal	72 mA h g^−1^	77%/9000	[136]
13	MXene/NiSe_2_	Hydrothermal	531 F g^−1^	—————‐	[137]
14	MXene/1T‐WS_2_	Gel electrolyte	113 F g^−1^	85%/5000	[138]

### 4.3 MXene and 2D Layered Double Hydroxide Composites

Recently, 2D transition metal layered double hydroxides (LDHs) have been attractive electrodes for energy devices due to their higher theoretical capacitance and low preparation cost.^[^ ^139^, ^140^ ^]^ Generally, LDHs exist in the form of [M_1‐y_^2+^ M_y_^3+^ (OH)_2_]^y+^ [A^m−^]_y/m_.zH_2_O, where M^2+^ and M^3+^ indicate the two‐ and three‐valent transition metals. Here, *y* denotes the ratio of M^2+^ (total metal atoms) and A^m−^ (anion charge) in 2D material and OH sites.^[^ ^141^, ^142^, ^143^ ^]^

There are disadvantages to a pure LDH nanostructure for practical uses such as low stability, surface re‐stacking, limited electrolyte transfer rate, cycle stability, and rate performance.^[^ ^144^, ^145^, ^146^ ^]^ As a result, 2D LDH with 2D MXene nanocomposite is expected to overcome the drawbacks of 2D LDH and enhance supercapacitor performance. The main crucial field of research is the double TMO composition in LDH and the composition–weight ratio of LDH to MXene for supercapacitor performance. MXene/LDH 2D/2D nanocomposite has been of great interest because of the plentiful surface functional groups of MXene. Hence, the ability of an LDH layer to settle over the MXene surface in the form of nanosheets like nanostructures.^[^ ^147^ ^]^ The electronic interaction between MXene and LDHs results in enhanced conductivity, active redox reaction, mechanical robustness, and cyclability. The LDH coated over the MXene nanosheet reduces interfacial resistance considerably to quicken electron transport and progress the kinetics of the electrochemical reaction. Thus, 2D/2D MXene/LDH has displayed excellent performance by surmounting the shortcomings of individual structures and improving aggregation and volume expansion during supercapacitor testing.^[^ ^148^, ^149^ ^]^ Importantly, MXene/LDH preserves the individual nanostructures while combined with each other, which is extremely essential for supercapacitor applications.^[^ ^150^, ^151^, ^152^, ^153^, ^154^ ^]^ To enhance the supercapacitor performance of LDH, more research groups have been devoted toward enhancing their nanostructures. Therefore, in this section, we will discuss LDH and MXene as a potential nanocomposite for supercapacitors. The MXene/CoAl‐LDH electrode was synthesized electrostatically for supercapacitor application.^[^ ^155^ ^]^ CoAl‐LDH content in the composites increased from 70 to 90 wt.%; hence, the stacking order for both MXene and CoAl‐LDH significantly decreased, which may be due to the electrostatic attraction among them. The optimized MXene/CoAl‐LDH electrode has a capacity of 2472 C cm^−3^ with stability of 70%. The full‐cell device obtained an energy density and power density of 30 W h kg^−1^ and 85 W h L^−1^, respectively, with a retention of 94% recorded over 30000 cycles.

NiMn‐LDH nanosheets/MXene sheets were prepared using a chemical method.^[^ ^156^ ^]^ From FE‐SEM images, it was observed that LDH sheets were coated uniformly over the MXene surface. The prepared NiMn‐LDH/MXene showed a capacitance of 1575 F g^−1^ with stability of 90% over 10000 cycles. The fabricated supercapacitor activated carbon//NiMn‐LDH/MXene device delivered a higher capacity of 169 F g^−1^ and cycling stability of 91% recorded at 10000 cycles, and also exhibited a higher energy/power density of 0.74 W h kg^−1^/126 kW kg^−1^. MXene/Ni‐Co‐LDH was prepared using an electrodeposition method.^[^ ^157^ ^]^ The capacitance of MXene/Ni‐Co‐LDH achieved 983 F g^−1^ at 2 A g^−1^ and cyclability of 76% (at 5000 cycles). Additionally, the 2D nanosheet of NiMn‐LDH stopped the re‐stacking issue in MXene, increasing the supercapacitor performance with fast electron/ion transportation. The supercapacitor device achieved a high an energy density of 36 W h kg^−1^ and power density of 1 kW kg^−1^. A porous NiFe‐LDH/MXene sample was synthesized by the hydrothermal method.^[^ ^158^ ^]^ Here, electrochemical performance improved through the nanosheet nanostructure and conductive network of NiFe‐LDH and MXene, which can fast‐track electron transport. SEM images of NiFe‐LDH/MXene composites showed that MXene and LDH form interconnected network structures with a thickness of 10–20 nm. The electrochemical analysis showed redox activity in the cyclic voltammetry and galvanostatic charge‐discharge curves, higher current density and charge/discharge time compared to MXene, and higher capacitance of 720 F g^−1^ with stability of 86% (1000 cycles).

**Figure 7:**
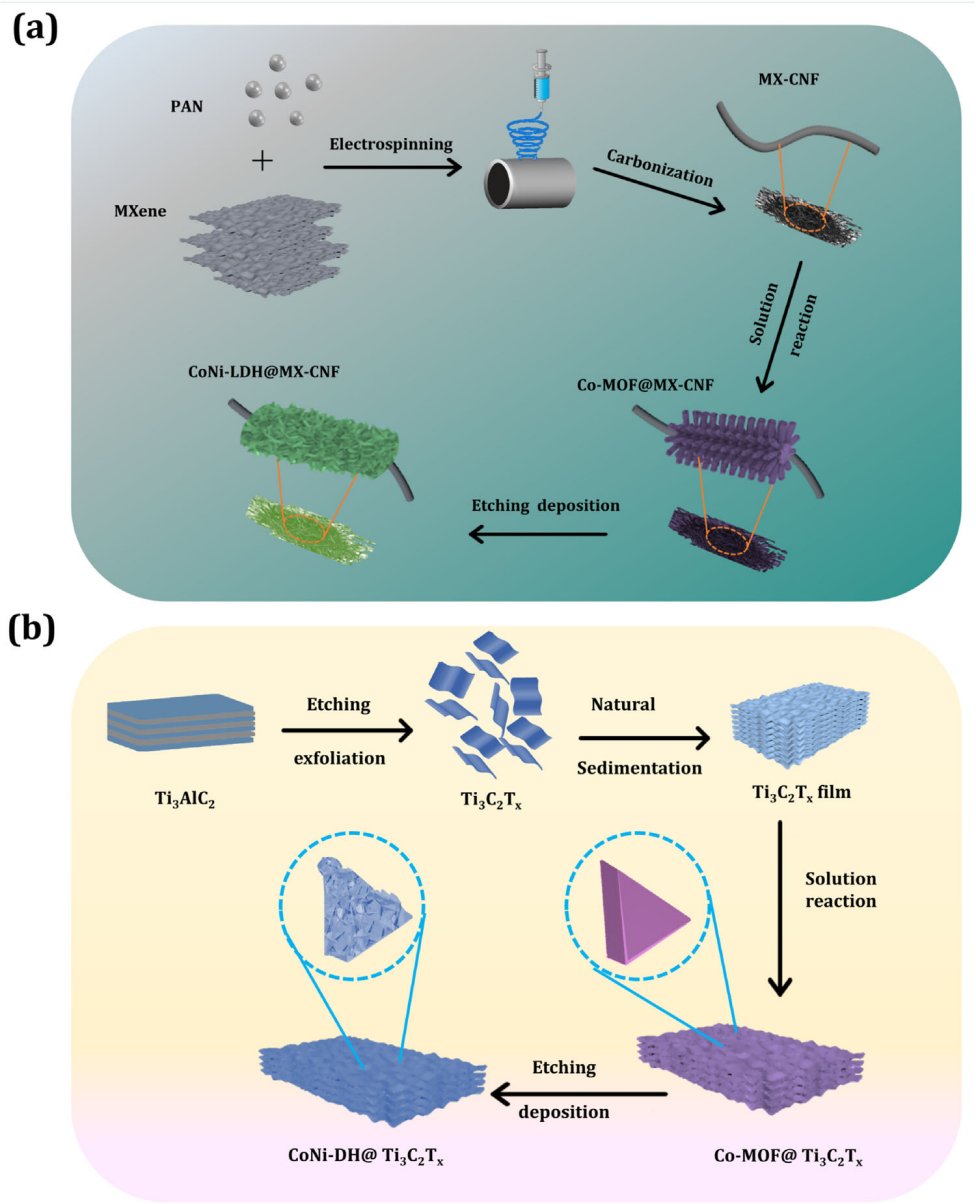
Synthesis of MXene and layered double hydroxide‐based nanocomposite using solution reaction and etching methods. a) Schematic illustration of preparation of cobalt−nickel layered double hydroxides (CoNi‐LDH) on MXene‐carbon nanofibers (MX‐CNF). MXene and carbon nanofibers (MX‐CNF) obtained by electrospinning, followed by the formation Co‐MOF@MX‐CNF and CoNi‐LDH@MX‐CNF electrodes using solution and etching reaction methods (Re‐draw from reference paper).^[^ ^159^ ^]^ b) Schematic illustration of cobalt‐nickel double hydroxide@MXene (CoNi‐DH@MXene) electrode synthesis using room temperature solution and etching methods (Re‐draw from reference paper).^[^ ^160^ ^]^

Co‐MOF@MX‐CNF was prepared using the electrospinning method as shown in **Figure** **7**a.^[^ ^159^ ^]^ The prepared CoNi‐LDH@MX‐CNF exhibits capacity of 996 F g^−1^ at 1 A g^−1^ and 78% retention over 3000 cycles. The asymmetric supercapacitor device CoNi‐LDH@MX‐CNF//activated carbon electrode delivered an energy density of 48.4 W h kg^−1^ and a power density of 499 W kg^−1^ with 78% retained at 3000 cycles. The significant contribution of this work is illustrated schematically in Figure 7a, where MXene is combined with carbon nanofibers via electrospinning, leading to the formation of free‐standing, flexible MXene‐carbon electrodes for energy storage devices. This is followed by the direct coating of CoNi‐LDH nanosheet nanostructures onto the MXene‐carbon composite using a low‐cost, simple, room temperature dip‐coating method. The CoNi‐LDH nanosheets increased the surface area and provided more active sites while reducing the interfacial resistance between the MXene‐carbon composite and CoNi‐LDH, leading to improved supercapacitor performance. This paper highlights DFT calculations that confirm the strong chemical bond interaction between MXene and CoNi‐LDH, enabling fast charge transport, higher conductivity, and enhanced long‐term stability of the nanocomposite. Overall, the DFT results are in strong agreement with the experimental findings.

The CoNi‐DH/MXene nanocomposite was created using an etching–deposition–growth process as shown in Figure 7b.^[^ ^160^ ^]^ Electrochemical analysis revealed that the CoNi‐DH/MXene nanocomposite showed capacitance of 919 F g^−1^ and stability of 89% over a 5000‐cycle scan. The full‐cell device, CoNi‐DH/MXene//activated carbon, was fabricated and exhibited specific capacitance and stability in the order of 165 F g^−1^ (at 1 A g^−1^) with 91% retention (3000 cycles), and delivered an energy and power density of 58 W h kg^−1^ and 0.8 kW kg^−1^, respectively. The prepared CoNi‐LDH electrode design on the MXene surface had a higher surface area and exposed active sites of the CoNi‐LDH active materials to enhance cyclic voltammetry and galvanostatic charge‐discharge redox current and charging/discharging time. This paper explores a single‐step synthesis method designed to create hierarchical cobalt–nickel double hydroxide nanoarrays on the MXene surface for use in supercapacitors. The advantage of this synthesis method includes a room temperature process with no structural direction agent and not requiring any expensive instruments. By using a room temperature process, the interconnected ultrathin CoNi‐LDH nanosheets were uniformly wrapped on the MXene surface, which enhanced the ion diffusion rate and electrolyte penetration depth in the interior as well as more redox reaction active sites. Therefore, Figure 7b highlights the selection of a simple and straightforward synthesis method for MXene‐LDH‐based nanocomposites at room temperature.

MXene and NiCo‐LDHs electrodes were prepared by self‐assemble method.^[^ ^161^ ^]^ SEM images of MXene/NiCo‐LDHs showed a well‐aligned, layered structure. The prepared MXene/NiCo‐LDHs electrode obtained capacitance of 1207 F g^−1^ and stability of 93% (5000 cycles). The prepared electrode showed a maximum an energy, and power density of ≈571 W kg^−1^ and 107 W h kg^−1^, respectively. MXene/NiAl was synthesized using a filtration method.^[^ ^162^ ^]^ From electrochemical data analyses, the QD‐MXene/NiAl‐LDHs electrode exhibited specific capacitance and high an energy, and power density of 2010 F g^−1^, 100 W kg^−1^, and 299 W kg^−1^, respectively, with stability of 94% (10000 cycles).

DFT calculations showed the effective adsorption of OH^−^, which enhanced supercapacitor performance. MXene/PDDA/NiAl‐LDHs were synthesized by electrostatic attraction.^[^ ^163^ ^]^ SEM images of MXene/PDDA/NiAl‐LDHs showed that each individual component alternates with one another, with a layer separation distance of ≈5–10 nm. Half‐cell data analysis of MXene/PDDA/NiAl‐LDHs showed high specific capacitance and cycling stability of 1825 F g^−1^ and 99%, after 5000 scans. NiCoFe‐LDH was coated over MXene via the hydrothermal method.^[^ ^164^ ^]^ The SEM and TEM results of the prepared NiCoFe‐LDH nanocomposite electrode confirmed that NiCoFe‐LDH nanosheets had grown over the MXene nanosheet. BET analysis confirmed that the NiCoFe‐LDH/MXene electrode (109 m^2^ g^−1^, 0.200 cm^3^ g^−1^) has higher surface area and is porous (3 nm), greater than for the individual electrodes. Electrochemical testing found NiCoFe‐LDH/MXene nanocomposite to have an excellent capacity of 1990 F g^−1^ and stability of 84% over 5000 continuous scans. The NiCoFe‐LDH/MXene//RGO asymmetric device delivered an energy density of 54 W h kg^−1^ and power density of 895.1 W kg^−1^. The interconnected MXene/LDH nanocomposite could be demolished during fast electrolyte ion adsorption and desorption. Thus, emerging high‐performance 2D MXene nanocomposite materials for supercapacitor applications necessitate careful evaluation of the LDH and MXene materials as well as a suitable interbreeding technique. **Table** **3** summarizes the reports for various 2D MXene/2D LDH nanocomposites with high‐performance supercapacitors.

**Table 3:** Electrochemical performance of MXene/LDH‐based supercapacitors.

Sr. No.	Material	Synthesis methods	Specific capacitance	Stability/Cycles	Refs.
1	MXene/CoAl‐LDH	Electrostatic	2472 C cm^−3^	70%/—‐	[155]
2	MXene/NiMn‐LDH	Chemical bond	1575 F g^−1^	90%/10000	[156]
3	MXene/NiCo LDH	Electrodeposition	983 F g^−1^	76%/5000	[157]
4	MXene/NiFe‐LDH	Hydrothermal	720 F g^−1^	86%/1000	[158]
5	MXene/CoNi‐LDH	Electrospinning	996 F g^−1^	78%/3000	[159]
6	MXene/CoNi‐DH	Etching	919 F g^−1^	89%/5000	[160]
7	MXene/NiCo‐LDHs	Electrostatic	1207 F g^−1^	93%/5000	[161]
8	MXene/NiAl‐LDH	Filtration	2010 F g^−1^	94%/10000	[162]
9	MXene/NiAl‐ LDH	Electrostatic	1825 F g^−1^	99%/5000	[163]
10	MXene/NiCoFe‐LDH	Hydrothermal	1990 F g^−1^	84%/5000	[164]

## 5 Conclusion and Future Perspectives

This review focused on 2D MXene and 2D materials such as TMO, TMD, and LDH, and their applications in supercapacitors. Particularly, the review introduced the preparation methods, nanostructures, and electrochemical properties of MXene and 2D materials. Additionally, the progress of MXene and 2D MXene/2D nanocomposite materials for supercapacitor applications built to date has been covered in this review and described in terms of morphology, structures, and functional termination groups. MXenes can function as highly conductive substrates, enabling quicker ion and electron transport. To stop the MXene sheets from re‐stacking, TMOs might be placed between them.^[^ ^165^, ^166^ ^]^ Supercapacitor electrode materials can be made from synthetic MXene and 2D materials nanocomposites, which have better electrical conductivity, a wider surface area, excellent active sites and functional groups, and strong structural stability.^[^ ^167^, ^168^ ^]^ It should be highlighted, nevertheless, that MXene and 2D materials nanocomposite technology is still in its infancy and more investigation is needed to determine how this material is developed at the device level. There is also the fantastic chance to create innovative MXene and 2D materials, wherein challenges can include a simple and cost‐effective preparation method, restricted redox sites, stability, sluggish kinetics and diffusion rates, and tuning the nanostructure of the materials. The chemical composition, essential to understanding the formation and growth mechanisms, is still not revealed. Using in situ techniques (HR‐TEM, Raman, and XRD) throughout the electrochemical measurement process is crucial for conducting a thorough analysis of the interfacial redox reactions, intercalation and de‐intercalation, and electronic structures of MXene and 2D nanocomposite materials. Hybrid, symmetric, and asymmetric energy storage devices are still necessary for their real practical uses and there are still improvements to be made in the electrochemical properties of various electrolytes. MXene and 2D materials can be used in multivalent batteries, including sodium‐ and lithium‐ion batteries as well as an anode material (Al^3+^, Mg^2+^, or Zn^2+^). More research is required to achieve these goals. MXene and 2D materials have shown high potential for supercapacitor applications in recent years. Thanks to its unique electrochemical and structural characteristics, the nanocomposite exhibits heightened inventiveness and superior electrochemical performance, rendering it highly suitable for diverse applications in fields like flexible and wearable electronics as well as supercapacitors. Furthermore, its versatility extends to applications in medicine, aquatic purification, CO_2_ capture, and beyond. Ultimately, we believe this study lays a solid groundwork for the advancement of novel 2D MXene and 2D materials nanocomposites, offering promising pathways for innovation within the energy storage domain.

## Conflict of Interest

The authors declare no conflict of interest.

## Data Availability

The data that support the findings of this study are available from the corresponding author upon reasonable request.
